# Cellular Activity of CQWW Nullomer-Derived Peptides

**DOI:** 10.1021/acsomega.4c08860

**Published:** 2025-02-11

**Authors:** Steven Shave, Rebecka Isaksson, Nhan T. Pham, Richard J. R. Elliott, John C. Dawson, Julius Soudant, Neil O. Carragher, Manfred Auer

**Affiliations:** † Edinburgh Cancer Research, Cancer Research UK Scotland Centre, Institute of Genetics and Cancer, University of Edinburgh, Crewe Road South, Edinburgh EH4 2XR, U.K.; ‡ School of Biological Sciences, University of Edinburgh, The King’s Buildings, Edinburgh EH9 3BF, U.K.; § Department of Chemistry, University College London, 20 Gordon Street, London WC1H 0AJ, U.K.; ∥ College of Medicine and Veterinary Medicine, University of Edinburgh, Institute for Regeneration and Repair, 4-5 Little France Drive, Edinburgh EH16 4UU, U.K.; ⊥ Departamento de Farmacologia, Facultad de Medicina, Universidad Autónoma de Madrid, Calle Arzobispo Morcillo 4, Madrid 28029, Spain

## Abstract

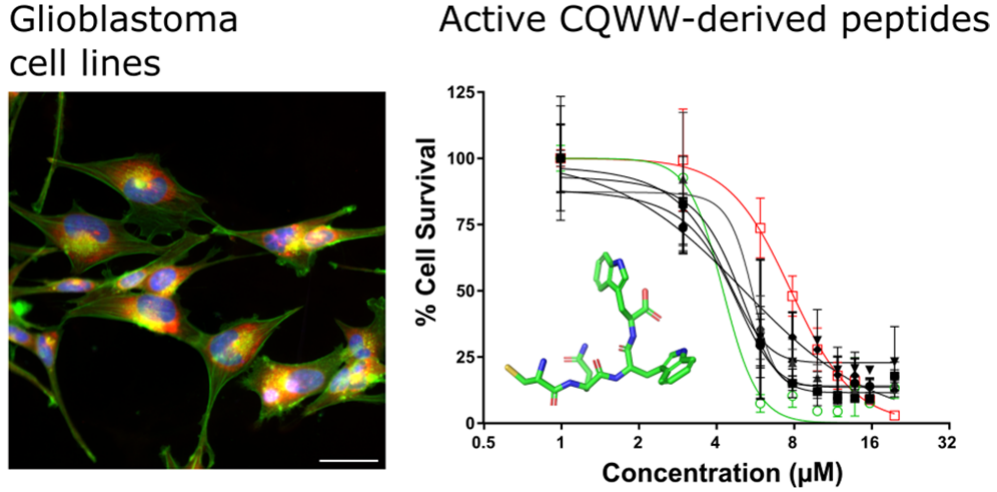

Analysis of observed protein sequences across all species within the UniProtKB/Swiss-Prot data set reveals CQWW as the shortest absent stretch of amino acids. While DNA can be found encoding the CQWW sequence, it has never been observed to be translated or included in manually curated sets of proteins, existing only in predicted, tentative sequences and in a single mature antibody sequence. We have synthesized this “nullomer” peptide, along with 13 derivatives, reversed, truncated, stereoisomers, and alanine-scanning peptides, conjugated to polyarginine stretches to increase cellular uptake. We observed their impact against a healthy neuronal line and six patient-derived glioblastoma cell lines spanning three clinical subtypes. Results reveal IC_50_ values averaging 4.9 μM for inhibition of cell survival across tested oncogenic cell lines. High-content phenotypic analysis of cellular features and reverse-phase protein arrays failed to discern a clear mode of action for the nullomer peptide but suggests mitochondrial impairment through the inhibition of GSK3 and isoforms, supported by observations of reduced mitochondrial stain intensities. With a recent increase in interest in nullomer peptides, we see the results in this study as a starting point for further investigation into this potentially therapeutic peptide class.

## Introduction

The term “nullomers” was introduced in 2007 to refer to DNA sequences absent from the human genome.^1^ These nullomers have subsequently been identified across a range of domains and species, finding uses as molecular barcodes in tamper-proof labeling of evidence from crime scenes^2^ and in the creation of homology models investigating genome evolution.^3^ Vergni and Santoni assigned sequences to a category named “high-order nullomers”.^4^ These are sequences that remain nullomers when mutations are introduced. In the same work, they analyzed nullomer occurrences in CpG islands, noting them to be present at a higher rate than expected but not high enough to suggest that nullomer formation is driven solely by natural selection. They stated nullomers had “their own peculiar structure and are not simply sequences whose CpG frequency is biased”.^4^ CpG island nullomer occurrences were, however, given as a potential explanation over natural selection by Acquisti et al.,^5^ who argued that the hypermutability of CpG islands contributes to rare sequences becoming nullomers, coupled with the fact that many in humans differ by only one residue, the role of mutation is strengthened in the localization and preservation of nullomers across species.^5^ Similarly, Georgakopoulos-Soares et al. use nullomers to build phylogenetic classifiers across vertebrates,^6^ while a wider study by Garcia et al. classifies 22 organisms across archaeota, bacteria, and eukaryotes using only the shortest nullomers from each genome.^7^ In an approach using codon-translated amino acids, Mouratidis et al.^8^ define sets of “quasi-prime” peptides or short peptide k-mers^9,10^ as being sequences found in only one species and propose using this data for species identification from biological samples. The idea of searching for absent peptide sequences appears in literature before this terminology referring to DNA was established, with Otaki et al. identifying 12,080 “zero-count” pentamer peptides from 1.5 million protein sequences obtained from public databases.^11^ While the absence of some pentamers may be explained by the low occurrence rates of their constituent amino acids, calculations accounting for amino acid propensities demonstrated that many should have been expressed in a theoretical genome. The opposite was also observed, with pentamers composed of low-propensity amino acids being unexpectedly present. Otaki et al. explained this through the evolutionary suppression of these peptide sequences,^11^ an explanation that is backed up by Tuller et al. through their discovery that many of the absent pentamer sequences are coded for in noncoding regions of the genome.^12^

Attempting to understand potential evolutionary pressure on nullomers, Navon et al. observed expected rates of peptide triplets occurring in the *E. coli* proteome, noting underexpression in only four peptide triplets: CMY, GPP, MWC, and WMC.^13^ While examination of triplets undoubtedly leaves out longer interesting sequences, Ung and Winkler identified triplets as occupying a special area of chemical space, possessing optimal ligand efficiency and proposing tripeptide motifs as being the optimal size molecule for biological signaling.^14^ Embedding these sequences in GFP and mntA reduced in vivo and in vitro expression levels not only of the proteins themselves but also their unmodified partners during coexpression. This is explained for the CMY and GPP triplets through observed interactions with ribosomal nucleotides A2062 and U2585, known for their involvement in ribosome stalling.^15,16^ They note that unlike in *E. coli*, all triplets are observed at expected rates when interrogating the human proteome.^13^ In 2019, Mittal et al. reported the counts of all dimer, trimer, tetramer, and pentamer peptides present within UniProtKB/Swiss-Prot, although no synthesis or biological testing was performed on newly discovered nullomer peptides.^17^ Later, the propensity of amino acid stretches in ordered versus intrinsically disordered proteins was explored by Mittal et al., leading to the identification of 36 unique tetramer peptides exclusively found within intrinsically disordered proteins^18,19^

Closely related to nullomer peptides, rarely observed amino acid stretches have also been studied by Capone et al., who assert that the minimal sufficient antigenic determinants of a protein could be encoded in just five rarely seen amino acids.^20^ Complementing this, Patel et al. noted that these rare sequences could be used to enhance antigen-specific immune responses when dosed alongside adjuvant vaccines.^21^ It was also noted by Koulouras et al. that human viruses rarely share human nullomers, facilitating host mimicry and immune evasion,^22^ an interesting example of this being highlighted by Silva et al. in their studies on the Ebola virus, identifying human nullomers which consistently appear in two viral Ebola proteins.^23^ Rare sequences were observed at higher than expected rates in proto-oncogenes by Trost et al.^24^ and Tsiatsianis et al.^25^ This points to a potential safety mechanism being inbuilt into these proto-oncogenes, facilitating natural identification and disposal and spurring interest in nullomer epitopes within immunology and oncology for the potential of these rare sequences to aid the immune system in the identification of cancerous cells. In 2012, an early stage drug discovery study by Alileche et al.^26^ synthesized pentamer peptide nullomers and discovered two peptides, NWMWC and the permutation WCMNW, that caused mitochondrial impairment in both normal cell lines and cancer cell lines.^26^ In a follow-up study, the lethal effect of these pentamers was further explored using the NCI-60 panel^27^ containing 60 cell lines derived from human cancers across nine different organs. Both NWMWC and WCMNW were lethal to a high fraction of oncogenic cell lines while not killing the majority of nononcogenic cell lines tested. Notably, the peptides were able to kill both drug-resistant and hormone-resistant prostate and breast cancer cell lines, as well as cancer stem cells,^28^ through a mechanism of mitochondrial impairment and ATP depletion.^26^ More recently, Ali et al. investigated WCMNW peptide activity in a triple-negative breast cancer mouse model using transcriptomics techniques to reveal the downregulation of key genes involved in the mitochondrial TCA cycle.^29^

Standard medicinal chemistry approaches including conjugation and derivatization of peptides to include non-natural amino acids, cyclization, and other stabilization strategies are now available to overcome many of the limitations present with the use of peptides as therapeutics, improving delivery pharmacokinetics and increasing proteolytic stability.^30,31^ Over 80 peptide drugs are currently marketed for a variety of diseases.^32,33^ This route from peptide to stable and efficacious treatment suggests the approach of using nullomers as a starting point to find new first-in-class therapeutics is both valuable and viable.

We developed our own implementation of a nullomer and rare sequence discovery algorithm named Aminonaut. This implementation written in Python allows easy integration into existing analysis pipelines and workflows. To facilitate reuse and open science, Aminonaut source code is available on GitHub (https://github.com/stevenshave/Aminonaut) and has also been added to the Python Package Index, allowing installation with a single command to nearly all modern Python environments. We applied Aminonaut to the February 2018 UniProtKB/Swiss-Prot database.^34,35^ This article documents our identification and initial biological evaluation of the tetramer nullomer peptide CQWW conjugated to a polyarginine sequence to ensure cellular uptake, with its striking absence begging the question: what specifically makes this sequence “forbidden” in vivo and what might be the biological effects of such a molecule?

## Results and Discussion

### Identification and Synthesis of CQWW

**Figure 1:**
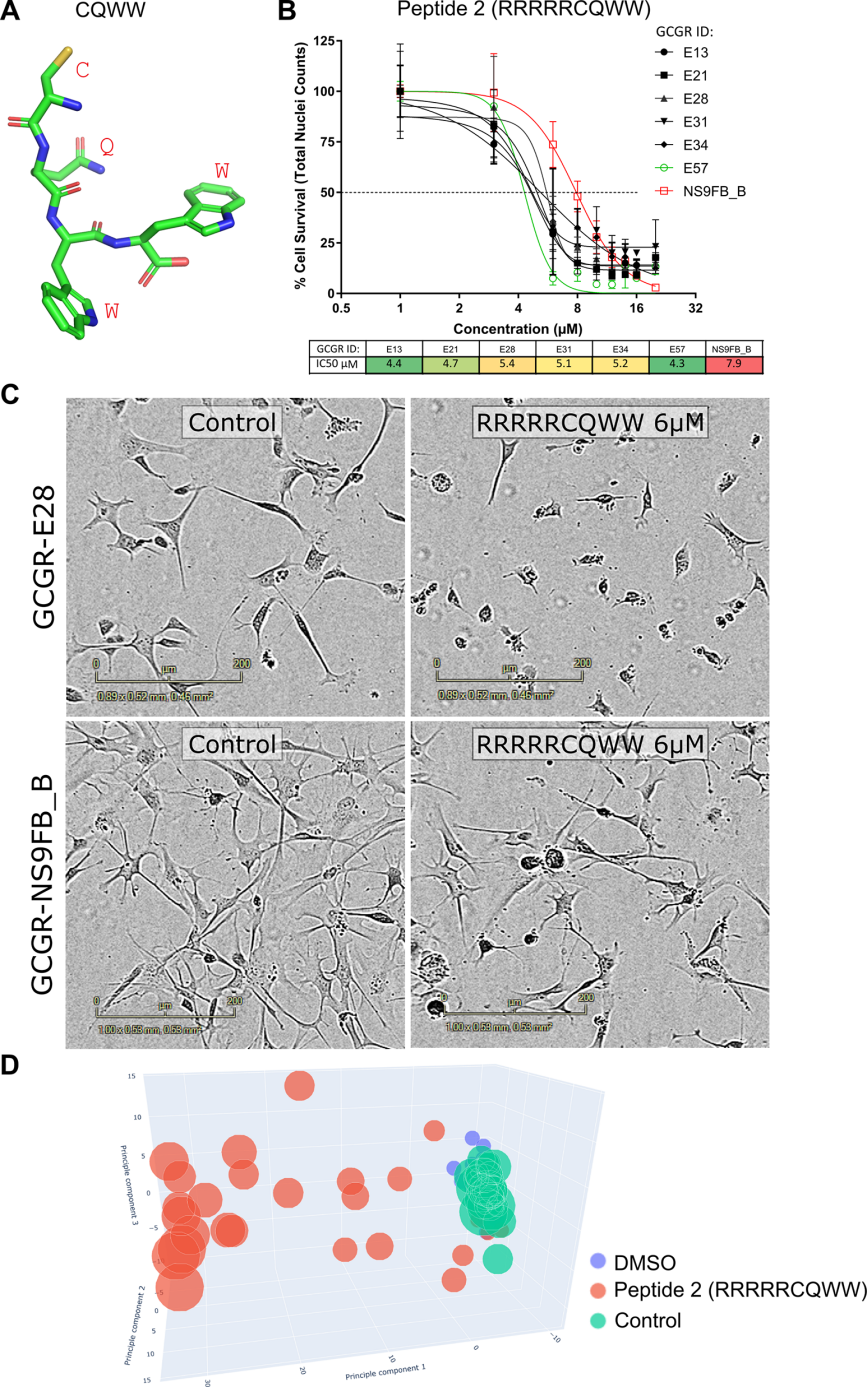
(A) 3D model of the CQWW peptide with residue labels shown in red. (B) Dose-dependent effect of peptide 2 (RRRRRCQWW) on cell survival across a range of cell lines, with fitted IC_50_ values shown below. (C) Representative live cell brightfield images of peptide 2 (RRRRRCQWW, 6 μM) against the glioblastoma stem cell line (GCGR-E28) versus normal neural stem cells (GCGR-NS9FB_B) after 3 h of incubation. (D) 3D principal component analysis (PCA 1, 2, and 3) from multiparametric cell painting image analysis of the GCGR-E13 cell line. A clear dose-dependent phenotypic profile with marker sizes denoting the concentration range of 1–16 μM is seen for peptide 2 (RRRRRCQWW, red) vs polyarginine control (RRRRR, green) and DMSO (blue).

We developed the Aminonaut package to specifically look for nullomer and rare protein sequences. Using a *k*-amino acid-sized window, this window is moved across protein sequences, counting occurrences of *k*-mers, where *k* is 2, 3, 4, 5, and 6. Further collation and statistical analysis are also built into the software. We used this software (see Methods) to examine nullomer and rare sequences within the UniProtKB/Swiss-Prot February 2018 release (see Supporting Information). Analysis of pentamer sequences reveals 86,261 nullomer sequences, representing 2.7% out of the total 3.2 million possible pentamers. In line with published results by Mittal et al.,^17^ we also identified the single missing tetramer, CQWW, cysteine–glutamine–tryptophan–tryptophan, from the collected tetramer counts. A BLAST search revealed the sequence is coded for in many proposed and hypothetical genomes,^36^ ranging from bacteria, oomycetes, to rotifers; however, they are absent from the carefully curated UniProtKB/Swiss-Prot releases, pointing to rare status and absence in proteomes captured in UniProtKB/Swiss-Prot. In 2021, the CQWW sequence was discovered in a human antibody immunoglobulin heavy chain in a study of the autoimmune disease Myasthenia gravis, whereby antibodies target neuromuscular proteins. Analysis of left and right sequence truncates of the CQWW tetrapeptide reveals CQW and QWW present 1251 and 1401 times, respectively, in the data set, well in line with expected occurrence rates. Interestingly, the CQW N-terminal trimer truncate peptide is found in patent literature for antibacterial use against *Listeria* and *Bacillus*.^37^ We did not find any biological effects noted for the QWW C-terminal trimer truncate in the literature or patents. CQ, QW, and WW dimers were observed at the expected rates. To find an explanation for the rare status of CQWW, we modeled the peptide (see Methods) to investigate if any intrinsic steric clashes or problematic conformational states were revealed (see Figure 1A). Aqueous solubility prediction was difficult as many online tools require query peptides to be longer than the CQWW tetramer, forcing us to turn to more traditional small-molecule solubility predictors. The SwissADME web service^38^ returns a range of predicted solubilities for the free peptide with poor agreement, ranging from 51 nM to 29 mM; however, these techniques are optimized for small molecules, adhering to more traditional medicinal chemistry rules.

The absent tetramer CQWW (peptide 1, Table S3) was synthesized using standard Fmoc peptide synthesis on a solid support (see Supporting Information). Chemical stability was observed via high-performance liquid chromatography (HPLC) (see Supporting Information and Figures S1 and S2 for results) over 72 h in phosphate buffered saline (pH 7.5), resulting in the formation of the disulfide bridged dimer form, confirmed by the addition of TCEP. This disulfide bridge-induced dimer formation was not observed upon the production and evaluation of the polyarginine conjugate RRRRRCQWW (peptide 2, Table S3). For this reason, and to improve peptide cell permeability,^39,40^ we produced subsequent peptides in this polyarginine conjugate form, following the approach taken by Alileche et al.^28^ of attaching a poly arginine chain to ensure cell penetration, all of which did not dimerize. In addition to peptide 2, we produced a d-stereoisomer form of RRRRRcqww (peptide 3, Table S3), a reversed form of RRRRRWWQC (peptide 4, Table S3), a reversed d-stereoisomer form of RRRRRwwqc (peptide 5, Table S3), alanine-scanning^41^ peptides (peptides 6–9, Table S3), and the N-terminal and C-terminal triplet truncates RRRRRCQW and RRRRRQWW (peptides 10 and 11, Table S3). Comparing the observed and expected counts based on either codon frequency or amino acid occurrence rates indicated that a majority of single amino acid mutations of CQWW appear at lower-than-expected frequencies. The highest count being for YQWW (207 occurrences) and the rarest (excluding glutamine) being CMWW (3 occurrences), see Table S1. Variations around the second position (glutamic acid) were found with lower counts than for other positions. To understand the implication of the cysteine residue, we produced low occurrence-count cysteine replacements (see Table S1) using methionine, histidine, and phenylalanine (peptides 12–14, Table S3). In addition to these synthesized peptides, we purchased a polyarginine control peptide, RRRRR (peptide 15, Table S3).

Peptides were tested in an automated image-based high-content^42^ cell painting^43,44^ assay that was originally developed to explore the mechanism of action of pharmacological and genetic perturbations within cells,^45^ as well as a live cell imaging assay against six patient-derived glioblastoma cell lines (GCGR-E13, GCGR-E28, GCGR-E21, GCGR-E57, GCGR-E31, and GCGR-E34), covering classical, mesenchymal, and proneural subtypes, respectively (see Methods and Figure 1B–D), along with a healthy human fetal neuronal stem cell line (GCGR-NS9FB_B).

Cell survival was quantified by nuclei counts from the Hoechst dye-stained nuclei and IC_50_ values calculated along with 95% confidence intervals (Figure 1B; see Table S2 for full details). Table 1 shows a summary of cellular IC_50_s derived from these nuclei counts.

**Table 1:**
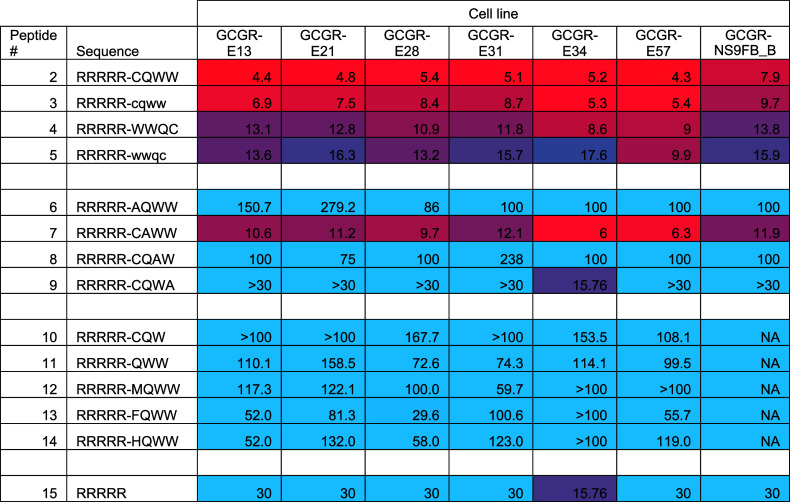
Cellular IC_50_s (μM) as Measured by Nuclei Counts for Nullomer and Derivative Peptides^a^

a Gradient between blue and red denotes high to low IC_50_, respectively. Peptides grouped by derivatives (2–5), alanine-scanning peptides (6–9), rare derivative sequences (10–14), and finally control (15).

Alanine-scanning peptides (Table 1, peptides 6–9) clearly demonstrate tolerance for alanine replacement at the second glutamine position, with peptide 7 (RRRRRCAWW) retaining an average IC_50_ of 14.8 μM across cell lines, while alanine replacement in other positions results in IC_50_ values greater than 100 μM (apart from the GCGR-E21 cell line with an IC_50_ of 75.0 μM—see Supporting Information Table S2 for full IC_50_ listings). The phenotypic effect of the peptides was visualized by live cell imaging (Figure 1C, IncuCyte live cell imager) and also quantified via image analysis of cells labeled with the cell painting reagents (see Supporting Information) followed by image analysis using Cell Profiler (cellprofiler.org). Multiparametric outputs from Cell Profiler were processed using Phenonaut^46^ [v 2.0.3] (see Jupyter notebook ‘Phenonaut_processing_of_nullomer_data.ipynb’ in the Aminonaut repository for methods and code used). Figure 1D shows a 3D principal component plot with a strong, dose-dependent phenotype for the nullomer peptide 2 when compared with the control polyarginine peptide 15 (RRRRR). Peptide 2 also showed approximately 1.5- to 2-fold selectivity for reduction in nuclei counts for glioblastoma stem cells compared to normal neural stem cells (GCGR-NS9FB_B). Peptides 2, 5, and 7 were deemed to have the most merit for mode of action determination using reverse phase protein arrays (RPPAs) (see Supporting Information for methods). Analysis of gene networks showed no clear mode of action, apart from downregulation of GSK-3β (see Supporting Information), which, among other functions, is noted as crucial for mediation of mitochondrial function.^47−50^ Analysis of mitochondrial stain intensities captured during the cell painting assay reveals a dose-dependent reduction in intensity for all cell lines (see Supporting Information Figures S3 and S4).

## Methods

### Bioinformatics

With an abundance of literature algorithms published to identify nullomers in protein and peptide forms,^1,51−53^ we drew on their descriptions to create our own custom implementation, filling a gap in Python bioinformatics tools and better integrating with our existing codebase and workflows. This took the form of a Python (version 3.8.5) program utilizing the NumPy library (version 1.19.1) for efficient array operations. This program was further developed into a suite of tools for interrogation of the UniProtKB/Swiss-Prot database and is available under the open source MIT license as a source code repository at https://github.com/stevenshave/Aminonaut.

After downloading the XML version of the February 2018 UniProtKB/Swiss-Prot database,^34,35^ the find_nullomer_motifs.py program was run, passing as arguments the XML file, followed by an output CSV file, and finally, the length of peptides to be counted. This was run for lengths of 2, 3, 4, 5, and 6 amino acids, directing output to different CSV files for each length. The 5- and 6-mers were captured with a “.csv.gz” file extension, directing the program to apply gzip compression.

Peptides were synthesized using a standard Fmoc solid-phase peptide synthesis. Further details are given in Supporting Information.

Modeling of the CQWW peptide was achieved using the PEPstrMOD service^54^ with default settings, embedding the peptide between alanine dimers to model a sequence of eight amino acids representative of the CQWW peptide embedded within a protein. Removal of flanking alanine residues and visualization of the remaining CQWW peptide was achieved using PyMol (v 2.5.0a0).

Methods for cell culture and phenotypic profiling are detailed in the Supporting Information accompanying this article.

## Conclusions

In this article, we have demonstrated the steps taken in our identification of the short CQWW peptide nullomer conjugated to a polyarginine sequence and profiled its biological activity using live cell and high-content imaging along with proteomics analysis using RPPA. While mechanism-of-action analysis with RPPA was inconclusive, perturbation of GSK-3 isoforms (see Supporting Information) aligns with the mitochondrial activity noted by Alileche et al.^28^ and our observed concentration-dependent reduction in mitochondria stain intensity. Alanine scanning shows a clear tolerance for changes to the glutamine in position 2, with efficacy retained upon replacement with an alanine. Interestingly, the backward and d-stereoisomer sequences retain similar activity across cell lines. The use of peptides for fundamental biology and drug discovery is rapidly increasing due to the size of addressable and explorable peptide chemical space, ease of chemical manufacture or biological expression, and their massive molecular recognition potential. To this end, and with peptidic drugs making up to 7% of new US FDA approvals from 2015 to 2019,^55^ we are said to soon be facing “the coming peptide tidal wave”.^56^ While nullomers and other rare peptides are poorly understood, it is vital that the body of knowledge on these special sequences is expanded and their full potential explored in the search for new first-in-class therapeutics.

## Supporting Information Available

Methods for chemical synthesis, cell culture, phenotypic screening, and RPPA analysis (PDF)

## Abbreviations

ATP: adenosine triphosphate
BLAST: basic local alignment search tool
CSV: comma-separated values
DNA: deoxyribonucleic acid
Fmoc: fluorenylmethyloxycarbonyl
GFP: green fluorescent protein
GSK3: glycogen synthase kinase 3
HPLC: high-performance liquid chromatography
NCI: National Cancer Institute
PTC: peptidyl transferase center
RPPA: reverse phase protein array
TCA: tricarboxylic acid cycle
TCEP: tris(2-carboxyethyl)phosphine
US FDA: United States Food and Drug Administration
XML: extensible markup language
mntA: manganese ABC transporter protein A
