# Supplementary material for: Cellular Activity of CQWW Nullomer-Derived Peptides
Source: ACS Omega. 2025 Feb 11;10(7):6794–800. doi: 10.1021/acsomega.4c08860 (PMC11865978; doi:10.1021/acsomega.4c08860)
Supplement: Supplementary file 1 — ao4c08860_si_001.pdf [file ao4c08860_si_001.pdf]

Supporting information:

Cellular activity of CQWW nullomer-derived peptides

Steven Shave[1,2,‡,], Rebecka Isaksson[2,3,‡], Nhan T. Pham[1,2,4], Richard J.R. Elliott[1], John C. Dawson[1], Julius Soudant[1,5], Neil O. Carragher[1], and Manfred Auer[2,*]*

[1] Edinburgh Cancer Research, Cancer Research UK Scotland Centre, Institute of Genetics and Cancer, University of Edinburgh, Crewe Road South, Edinburgh, EH4 2XR, UK.

[2] School of Biological Sciences, University of Edinburgh, The King's Buildings, Edinburgh, EH9 3BF, U.K.

[3] Department of Chemistry, University College London, 20 Gordon Street, London WC1H 0AJ, U.K.

[4] College of Medicine and Veterinary Medicine, University of Edinburgh, Institute for Regeneration and Repair, 4-5 Little France Drive, Edinburgh EH16 4UU, UK.

[5] Departamento de Farmacologia, Facultad de Medicina, Universidad Autónoma de Madrid, Calle Arzobispo Morcillo 4, 28029, Madrid, Spain.

Data and software availability

Occurrence count data for 2,3,4,5, and 6-mers may be downloaded from the University of Edinburgh's DataShare service using the following DOI link:

https://doi.org/10.7488/ds/2797

Reference:

Shave, Steven. (2020). Peptide occurrences dataset from UniProt Feb 2018, [dataset]. University of Edinburgh, School of Biological Sciences, Institute of Quantitative Biology, Biochemistry and Biotechnology. https://doi.org/10.7488/ds/2797.

Software developed and used during this investigation is available in the public GitHub repository at:

https://github.com/stevenshave/NullomerPeptides

Table S1: Peptide occurrences around the CQWW template, showing mutation counts at each position. Data derived from the UniProt/Swiss-Prot Feb 2018 release. Numbers in brackets denote observed enrichment over expected from randomized human codons at expected rates and observed amino acid rates in UniProt/Swiss-Prot Feb 2018 release, respectively. Calculations performed using CodonCounter class in accompanying NullomerPeptides source package. NA = not applicable.

**X-QWW**		**C-X-WW**		**CQ-X-W**		**CQW-X**	
**Y-QWW**	207 (3.64, 7.64)	C-D-WW	73 (1.28, 4.11)	CQ-R-W	171 (0.50, 2.64)	CQW-I	141 (0.83, 2.03)
**R-QWW**	114 (0.67, 2.22)	C-G-WW	45 (0.40, 1.95)	CQ-A-W	146 (0.64, 1.51)	CQW-L	139 (0.41, 1.23)
**G-QWW**	111 (0.98, 11.69)	C-L-WW	43 (0.25, 1.37)	CQ-L-W	128 (0.37, 1.13)	CQW-E	123 (1.08, 1.56)
**S-QWW**	107 (0.631.74)	C-W-WW	32 (0.28, 8.98)	CQ-G-W	104 (0.46, 1.26)	CQW-G	91 (0.40, 1.10)
**T-QWW**	93 (0.82, 1.87)	C-A-WW	32 (1.13, 1.19)	CQ-S-W	91 (0.27, 1.18)	CQW-V	88 (0.39, 1.10)
**L-QWW**	89 (0.52, 0.99)	C-V-WW	31 (0.27, 1.39)	CQ-T-W	78 (0.34, 1.25)	CQW-A	76 (0.33, 0.79)
**E-QWW**	86 (1.51, 1.37)	C-S-WW	31 (0.18, 1.44)	CQ-Q-W	77 (0.68, 1.68)	CQW-S	75 (0.22, 0.97)
**A-QWW**	81 (0.71, 1.06)	C-R-WW	28 (0.16, 1.55)	CQ-P-W	74 (0.33, 1.34)	CQW-D	69 (0.61, 1.08)
**V-QWW**	77 (0.68, 1.21)	C-T-WW	25 (0.22, 1.44)	CQ-D-W	73 (0.64, 1.14)	CQW-C	61 (0.54, 3.79)
**I-QWW**	75 (0.88, 1.36)	C-F-WW	24 (0.42, 1.91)	CQ-K-W	63 (0.55, 0.93)	CQW-K	57 (0.50, 0.84)
**D-QWW**	63 (1.11, 1.24)	C-C-WW	19 (0.33, 4.24)	CQ-E-W	61 (0.54, 0.77)	CQW-T	54 (0.24, 0.86)
**P-QWW**	62 (0.54, 1.41)	C-P-WW	13 (0.11, 0.85)	CQ-V-W	57 (0.25, 0.71)	CQW-R	53 (0.16, 0.82)
**K-QWW**	53 (0.93, 0.98)	C-Y-WW	12 (0.21, 1.26)	CQ-I-W	47 (0.28, 0.68)	CQW-F	51 (0.45, 1.13)
**N-QWW**	43 (0.76, 1.14)	C-K-WW	12 (0.21, 0.63)	CQ-H-W	44 (0.39, 1.66)	CQW-Y	41 (0.36, 1.20)
**Q-QWW**	40 (0.70, 1.10)	C-I-WW	12 (0.14, 0.62)	CQ-F-W	40 (0.35, 0.89)	CQW-Q	41 (0.36, 0.89)
**W-QWW**	38 (1.34, 3.74)	C-E-WW	10 (0.18, 0.46)	CQ-N-W	39 (0.34, 0.82)	CQW-P	37 (0.16, 0.67)
**M-QWW**	24 (0.84, 1.07)	C-N-WW	8 (0.14, 0.61)	CQ-Y-W	37 (0.33, 1.09)	CQW-N	32 (0.28, 0.67)
**H-QWW**	19 (0.33, 0.53)	C-H-WW	5 (0.09, 0.68)	CQ-C-W	35 (0.31, 2.17)	CQW-M	15 (0.26, 0.53)
**F-QWW**	19 (0.33, 0.90)	C-M-WW	3 (0.11, 0.38)	CQ-M-W	6 (0.11, 0.21)	CQW-H	6 (0.05, 0.23)
**C-QWW**	0 (NA, NA)	C-Q-WW	0 (NA, NA)	CQ-W-W	0 (NA, NA)	CQW-W	0 (NA, NA)

Note on observed/enrichment rates

If a purely random arrangement of four amino acids is taken, then given the number of protein sequences in UniProt/Swiss-Prot Feb 2018 release and the length of all sequences, we might expect to see each unique tetramer 1,231 times. However, not all amino acids have a consistent number of DNA codons coding for them. Considering human DNA codon frequencies, we would expect to observe the CQWW sequence 47 times in a dataset as large as UniProt/Swiss-Prot Feb 2018 release. Extracting amino acid occurrence frequencies from UniProt/Swiss-Prot Feb 2018 release indicates that we would expect to see CQWW 12 times.

Table S2: Peptide IC_{50}s (µM), calculated from nuclei counts, shown with 95 % confidence intervals.

Peptide	GCGR-E13 IC_{50} µM (95%CI)	GCGR-E21 IC_{50} µM (95%CI)	GCGR-E28 IC_{50} µM (95%CI)	GCGR-E31 IC_{50} µM (95%CI)	GCGR-E34 IC_{50} µM (95%CI)	GCGR-E57 IC_{50} µM (95%CI)	GCGR-NS9FB_B IC_{50} µM (95%CI)
**#2 RRRRRCQWW**	4.4 (3.7-5.1)	4.8 (4.1-5.5)	5.4 (4.6-6.3)	5.1 (4.0-6.3)	5.2 (4.2-6.2)	4.3 (wide)	7.9 (7.4-8.4)
**#3 RRRRRcqww**	6.9 (6.0-7.8)	7.5 (6.8-8.1)	8.4 (7.5-9.2)	8.7 (7.6-9.9)	5.3 (4.0-6.4)	5.4 (4.9-5.9)	9.7 (9.2-10.3)
**#4 RRRRRWWQC**	13.1 (10.6-17.0)	12.8 (11.5-14.3)	10.9 (9.5-12.4)	11.8 (9.9-13.8)	8.6 (7.4-9.7)	9.0 (8.1-9.9)	13.8 (13.0-14.6)
**#5 RRRRRwwqc**	13.6 (12.3-15.3)	16.3 (15.0-18.0)	13.2 (12.4-14.0)	15.7 (13.4-20.7)	17.6 (15.6-21.7)	9.9 (9.2-10.5)	15.9 (14.9-17.1)
**#7 RRRRRCAWW**	10.6 (9.4-11.8)	11.2 (10.6-11.9)	9.7 (9.1-10.2)	12.1 (11.0-13.3)	6.0 (5.4-6.6)	6.3 (5.9-6.7)	11.9 (11.5-12.3)
**#9 RRRRRCQWA**	>30 (N/A)	>30 (N/A)	>30 (N/A)	>30 (N/A)	15.8 (12.0-23.1)	>30 (N/A)	>30 (N/A)

Chemistry Methods

General

All chemicals and solvents used in the synthesis of peptides were purchased from commercial sources and were used without further purification. The amino acids used had the following protection groups: Fmoc-Arg(Pbf)-OH, Fmoc-Cys(Trt)-OH, Fmoc-Gln(Trt)-OH, Fmoc-Trp(Boc)-OH, Fmoc-D-Cys(Trt)-OH, Fmoc-D-Gln(Trt)-OH, Fmoc-D-Trp(Boc)-OH, Fmoc-Ala-OH, Fmoc-Met-OH, Fmoc-Phe-OH, and Fmoc-His(Trt)-OH. The solid support was TentaGel S Rink amide resin, purchased from Rapp Polymere (loading 0.22 mmol/g; S30023, 90 µm).

Purification of synthesized peptides was performed using preparative HPLC: Agilent 1100 series HPLC system with two preparative pumps (G1361A), preparative autosampler (G2260A), MWD detector (1365G), and fraction collector Prep-FC (G1364B). Columns: Agilent Prep C18 (41391-102), 21.2 x 150 mm, and Waters X-bridge Prep OBD, 18 x 100 mm. Solvent: H_2O (A) with acetonitrile (B) comprising 0.5% TFA or 0.5% formic acid. Flow rate of 20 mL/min (Agilent Prep C18) or 10 mL/min (Waters X-bridge Prep OBD).

Purity of the obtained peptides was determined using analytical HPLC: Agilent 1100 series HPLC system with quaternary pump (G1311A), degasser (G1322A), well plate autosampler (G1367A), FLD detector (G1321A), and DAD detector (G1315B). Column Zorbax SB-C18, 4.6×150 mm, 3.5 µm. Solvent: H_2O (A) with acetonitrile (B) comprising 0.1% TFA. Gradient used: 0 – 5% B; 5 – 24 min, 5 – 100% B, 24 – 26 min, 100% B; 26 – 27 min, 100 – 5% B; 27 – 30 min, 5% B. Flow rate of 0.8 mL/min.

The peptide mass was confirmed using Thermo Scientific LTQ XL Linear Ion Trap Mass Spectrometer with positive electrospray ionization mode.

Peptide synthesis

Peptides were synthesized using standard Fmoc solid phase peptide synthesis (SPPS), following this general protocol: The TentaGel S Rink amide resin was conditioned in dichloromethane (DCM) for 30 min. Fmoc deprotection was achieved by agitating the resin in 20% piperidine in dimethyl formamide (DMF; 2×20 min), after which the resin was washed repeatedly with DMF (6×1 min). The amino acid to be coupled (3.0 eq.), 1-[bis(dimethylamino)methylene]-1H-1,2,3-triazolo[4,5-b]pyridinium 3-oxide hexafluoro-phosphate (2.8 eq.; HATU), and *N*,*N*-diisopropylethylamine (5.0 eq.; DIPEA) were dissolved in DMF and added to the resin. After gentle agitation for 20 min, the resin was washed with DMF (6×1 min) and the coupling step was repeated. The resin was then again washed with DMF (6×1 min) and the Fmoc protection group was removed with 20% piperidine in DMF (2×20 min). The next coupling was performed using the same process until the desired peptide had been obtained. The resin was Fmoc deprotected, washed with DMF (6×1 min) and subsequently with DCM (15 mL) before it was dried using vacuum. Reagent K (trifluoroacetic acid [TFA, 82.5%], water [5%], thioanisole [5%], phenol [5%], and 1,2-ethaneditiol [2.5%]) was prepared fresh, added to the resin, and the mixture was agitated for 1.5-2 h. The resin was filtered off and the peptide was precipitated from the solution by adding cold diethyl ether. The solid was collected as a pellet after centrifuging for 10-30 min (at 4000 g). The pellet was resuspended in cold diethyl ether and centrifuged again, this process was repeated a total of five times. The pellet was subsequently dissolved in H_2O:acetonitrile (50:50) and freeze-dried before the peptide was purified on preparative HPLC. For peptides containing cysteine the peptide was dissolved in H_2O:acetonitrile (50:50) and treated with a five-fold excess of tris(2-carboxyethyl)phosphine (TCEP) for 2 h before the solution was freeze dried and purified.

<u>Stability assessment of CQWW</u>

The stability of Peptide 1 (CQWW) was assessed in 1× PBS with 0.5 % EtOH via HPLC at 220 nm (Agilent 1100). After 24 hours, almost all of Peptide 1 had dimerized as shown in Figure S1. Dimer formation was confirmed via mass spectrometry (Thermo Scientific LTQ XL Linear Ion Trap Mass Spectrometer).

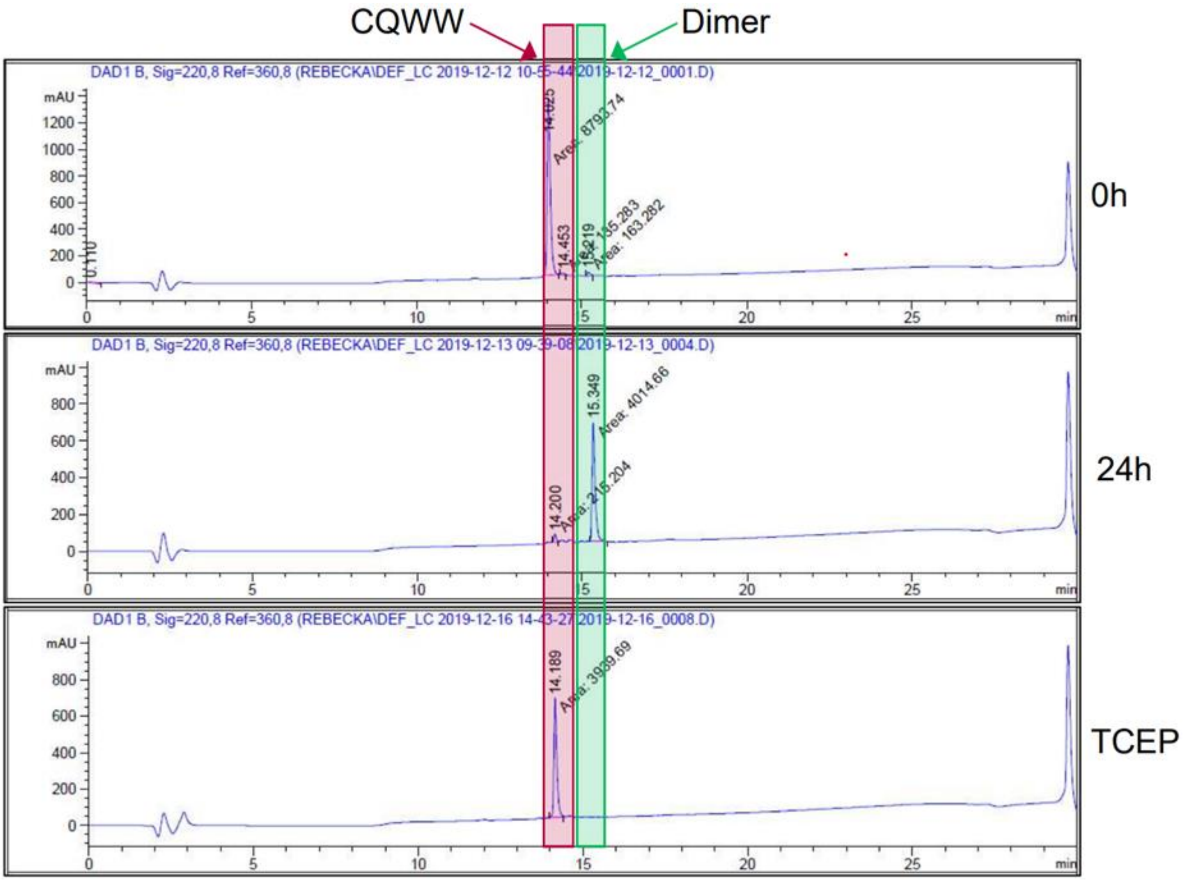

Figure S1 - Peptide 1 dimerization. No (or negligible) dimer is present at the starting timepoint of 0 hr (top). Dimer peak formation is shown at the 24 hr timepoint (middle). With the addition of TCEP to ensure free sulfhydryl groups, no dimerization is observed after 24 hrs (bottom). Dimer presence also confirmed using mass spectrometry.

Figure S2 shows dimer formation as a function of time and temperature.

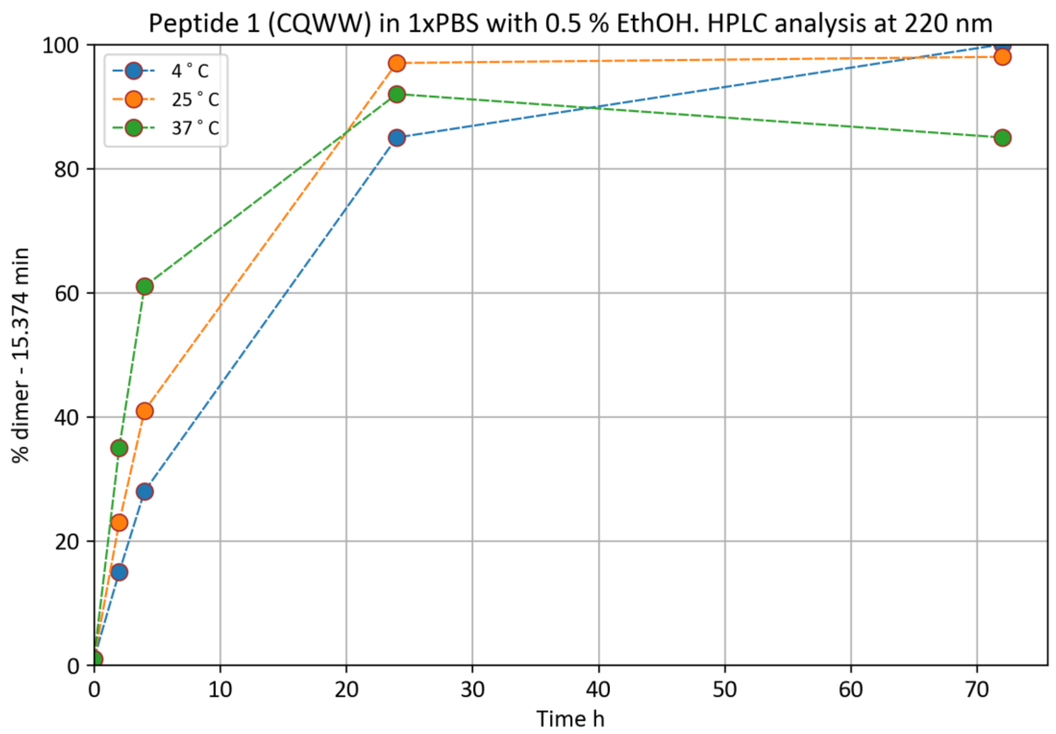

Figure S2 – Peptide 1 (CQWW) dimer formation as a function of time (0, 2, 4, 24, and 72 hours) and over three temperatures (4, 25, 37 °C).

Peptides synthesized

Table S3 – Summary of the yield and analytical data of synthesized peptides. *Peptide 15 was purchased with a C-terminal amide, from LifeTein at >95 % purity. Capital letters denote natural amino acids, small letters denote unnatural amino acids.

No	Compound	Yield [µmol]	HPLC purity at 220 nm (retention time)	Calculated mass	Observed mass; first fragmentation mass
**1**	CQWW	3.46	78% (14.07 min); 18% dimer (15.27 min)	620.24<br>1238.49	620.25<br>1239.33
**2**	RRRRRCQWW	4.27	>99% (13.51 min)	1401.65	1401.58; 701.33
**3**	RRRRRcqww	3.27	97% (13.32 min)	1401.65	1401.58; 701.33
**4**	RRRRRWWQC	4.67	96% (13.60 min)	1401.65	1401.67; 701.33
**5**	RRRRRwwqc	5.09	98% (12.99 min)	1401.65	1401.58; 701.33
**6**	RRRRRAQWW	3.27	98% (13.19 min)	1369.59	1369.58; 685.33
**7**	RRRRRCAWW	3.99	98% (13.33 min)	1344.60	1344.50; 672.83
**8**	RRRRRCQAW	3.11	98% (12.00 min)	1286.52	1286.50; 643.83
**9**	RRRRRCQWA	5.12	95% (11.97 min)	1286.52	1286.58; 643.83
**10**	RRRRRCQW	2.72	97% (12.02 min)	1215.44	1215.58; 608.33
**11**	RRRRRQWW	3.67	>99% (13.08 min)	1298.51	1298.58; 649.83
**12**	RRRRRMQWW	3.36	97% (13.56 min)	1429.70	1429.58; 715.33
**13**	RRRRRFQWW	4.63	>99% (14.00 min)	1445.68	1445.67; 723.42
**14**	RRRRRHQWW	3.55	98% (13.15 min)	1435.65	1435.67; 718.42
**15**	RRRRR*	NA	> 95%	797.95	NA

Biological methods

Cell culture

Human glioblastoma (GBM) stem cells (GCGR-E13, GCGR-E28, GCGR-E21, GCGR-E57, GCGR-E31, GCGR-E34) and normal foetal neural stem cells (GCGR-NS9FB_B) were obtained from the Glioma Cellular Genetics Resource (gcgr@ed.ac.uk). GBM stem cell line were cultured in DMEM/Ham's F12 media (Sigma D8437) supplemented with 7.25 mL Glucose (Sigma G8644), 5 mL MEM NEAA 100× (Gibco 11140-035), 800 µL BSA Solution 7.5% (Gibco 15260-037), 1 mL beta-mercaptoethanol 50 mM (Gibco 31350-010), 5 mL B27 Supplement 50× (LifeTech/Gibco 17504-044), 2.5 mL N2 Supplement 100×(LifeTech/Gibco

17502-048), EGF (Peprotech 315-09, 10 ng/mL), FGF (Peprotech, 100-18b, 10 ng/mL) . Cell growth and stemness was further supported by the addition of laminin extracellular matrix (Cultrex 3446-005-01, 4-10 mg/mL) to the growth media and to create pre-coated flasks/384w plates. Cells were cultured in T75 cm^2 flasks and passaged as follows: Glioma stem cells generally grow slowly (~60hrs doubling time, excepting GCGR-E57 cells ~24hrs) and lag phase was exacerbated by sparse seeding. Cell confluence is generally ~80-90% prior to passaging, whereby the culture media was aspirated, the cells washed (PBS) and Accutase solution (Sigma A6964, 1 mL) was added to detach the cells, which were then incubated at 37ºC for <5 minutes. Cells were resuspended in media and centrifuged for 3 minutes at 300× g. Resuspended cells were typically split 1:5, 1:8 (GCGR-E57 cells) and 1:2, 1:4 for the remainder of cell lines, depending on requirements. Cells were passaged every 4-7 days to maintain health and growth and discarded after 3 months, whereupon a fresh batch of cells would be thawed.

Reverse Phase Protein Array (RPPA)

Culture media was aspirated and cells washed with wash media. Accutase solution (1mL) was used to detach the cells which were then incubated at 37ºC for 5 minutes. 10 mL of wash media was added, and the cells were pipetted into a 15 mL conical tube, after which they were centrifuged for 3 minutes at 300× g to form a pellet. Supernatant was aspirated, and cells resuspended in 5 mL of wash media. A 10 μL sample of the cell suspension was pipetted onto the reading surface of a DeNovix CellDrop automated cell counter and the reading was used to dilute the suspension in complete media to 250,000 cells/mL. 2 mL of this suspension was added to each well of 6-well plates to seed plates at 500,000 cells per well. 2 plates of each cell type (GCGR-E13, GCGR-E21, GCGR-E28, GCGR-E31, GCGR-E34, GCGR-E57 and GCGR-NS9FB_B) were seeded, one each for two nullomer dosage

timepoints (3 h and 24 h). The cells were incubated in the 6-well plates overnight, and dosing was carried out in the following morning. Peptides 2, 5, and 7, were diluted in complete media to 3 μM, generating 30 mL of peptides 2 and 5 (2 mL for each cell line + each timepoint) and 15 mL peptide 7 due to limitations on the amount available which was enough for the 24-hour timepoint only. Water was used as a control. The media was aspirated, and 2 mL of the Nullomer solution was added, carefully pipetting against the side of the well to avoid disturbing the cell layer. The cell plates incubated at 37ºC for the duration of the respective timepoints.

The cell pellet preparation was performed on ice. After 3 and 24 hour incubation periods plates were removed from the incubator. The wells were washed with 2 mL of cold PBS each, which was promptly aspirated. 200 μL of Accutase was added to each well and incubated for 5 min at room temperature. 0.5 mL of wash media was added to the well and collected in the appropriate Eppendorf tube. Accutase cell detachment was repeated 2× to collect as many cells as possible and the tube was returned on ice. Once all cells were collected, the tubes were centrifuged in a pre-cooled (4 ºC) bench centrifuge at 300 x g for 3 minutes. The supernatant was aspirated and discarded. 1 mL of cold PBS was added to each tube and the pellet was resuspended using a pipette. The tubes were then centrifuged again using the same centrifuge settings. The supernatant was aspirated, and the pellets were stored at -80 ºC until ready for RPPA.

Frozen CLB1-Cell lysis buffer (Zeptosens-Bayer) was thawed immediately before use and 50 μL added tosample pellets, then vortexed for 10 seconds and left to incubate for 30 minutes at room temperature. The lysed samples were then centrifuged at 300 × g for 5 minutes. The supernatant was collected, and the pellet was discarded. To normalize samples for equivalent protein concentration prior to printing on RPPA, a Bradford assay analysis was performed and sample concentrations were adjusted to 1 mg/mL.

After protein concentration normalisation, dilution plates were prepared in a V-bottomed 96-well plate using CSBL1-spotting buffer (Zeptosens-Bayer) and CLB-1 in an 80:20 ratio. 20 μL of sample was added in row 1 with 180 μL CSBL1/CLB1 mixture. 60 μL, 40 μL and 20 μL of row 1 dilution were added to rows 2, 3 and 4 respectively, using the original CSBL1/CLB1 mixture to top the rows up to 80 μL. 40 μL of each sample was transferred to a 384-well plate in a pattern suited to the Zeptosens spotter set-up. A control plate was prepared with pre-optimised labelled BSA as a biological protein control. The 384-well plates were centrifuged at 200 × g for 5 minutes at room temperature. The slides were spotted using the GeSIM Nanoplotter. After drying for 2 hours, the slides were placed into blocking racks, and blocked for 1.5 hours using the ZeptoFog blocking station and BB1-blocking buffer (Zeptosens-Bayer). The slides were washed 3 times in distilled water for 2 minutes in the blocking racks, and then centrifuged at 200 × g for 5 minutes to dry. The slides were moved to Zeptocarriers and washed 3 times with 90 μL of CAB1 buffer (Zeptosens-Bayer). Once the primary antibody was made up, the CAB1 was aspirated, the antibody was added, and the plate was sealed and incubated overnight at an angle of 15 º. The primary antibody was aspirated and washed 3 times with CAB1 as before. The secondary antibodies were made up and the zeptocarriers were incubated in the dark for 2.5 hours at an angle of 15º. The antibody was removed and zeptocarriers washed 3 times with CAB1 as before, leaving the last CAB1 wash. The slides were scanned using the Zeptoreader.

To perform quality control on the Zeptosens platform, a Shapiro-Wilk test was performed on the intensity distribution of each dilution range to establish normality. The treated samples were converted to a fold difference by dividing the average of three technical repeats by the control value at each timepoint. Any values above five and below zero were considered artifacts and were removed. Fold difference was then transformed (Log_2 ratio) to create a symmetrical measure from which a heatmap was generated (see Figure S3) followed by

hierarchical clustering (One Minus Pearson Correlation and Average linkage method) using Morpheus (https://software.broadinstitute.org/morpheus/), grouped by treatment.

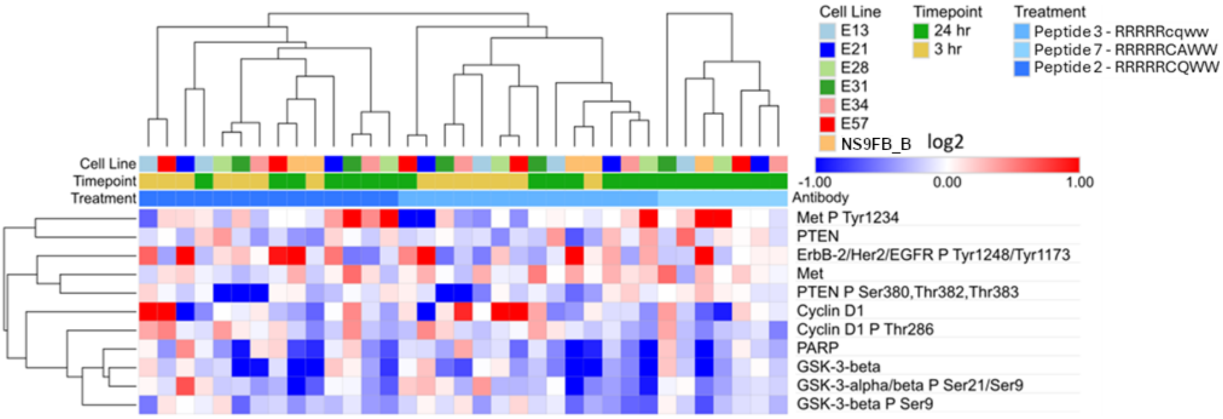

Figure S3 – Top 11 up/down regulated antibodies heatmap for peptides 3 (RRRRRcqww), 7 (RRRRRCAWW) and 2 (RRRRRCQWW) over a range of timepoints and cell lines, showing a downregulation of GSK-3.

Hierarchical clustering identified increased or decreased expression of certain signalling pathways, which were analysed further by network analysis (STRING database, string-db.org) to examine the broader impact of the treatments (Figure S4). Enrichment analysis (biological processes) indicated gene ontology terms associated with transmembrane RTK and Cell Surface Receptor Signalling, as well as ErbB signalling and regulation of mitochondrial depolarisation.

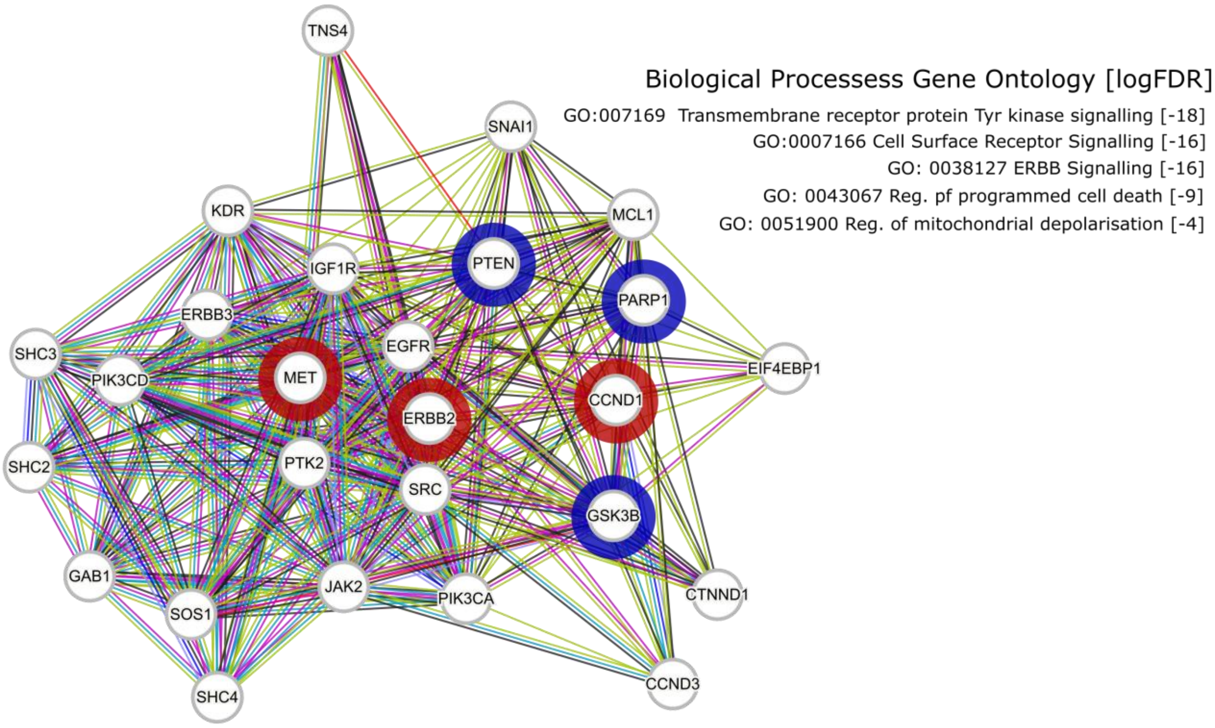

Figure S4 – Network analysis (STRING) showing protein interactions resulting from treatment with Peptide 2 (RRRRRCQWW). Indicated proteins in red (Met, ERBB2, CCND1) showed increased expression and blue (PTEN, PARP1, GSK3B) decreased expression with max number of interactors (1st, 2nd shell) <10. Top ranked Gene Ontology (GO terms) for biological processes is listed (top right) with Log[False Discovery Rate] indicated.

<u>High Content Phenotypic Screening assays</u>

Cell lines (glioma stem cell lines and normal foetal neural stem cells) were seeded on laminin (10 µg/mL) coated 384-well plates (Greiner Bio-One, 781091) at 1,000 cells/well and incubated overnight at 37°C and 5% CO_2. Aqueous peptide solutions (10 mM) were further diluted to 1 mM in water containing 10 % glycerol and 0.01 % TX-100. Cells were dosed using a D300 digital dispenser (Tecan) by 10-point dose response (30 µM – 1 µM) in triplicate and with aqueous glycerol/TX-100 as control. The cells were placed in an IncuCyte live cell imager (Essen BioScience) and imaged every three hours for 72 hours. Prior to fixation, the plates were removed from the IncuCyte and stained with Mitrotracker DeepRed (Invitrogen, M22426, 300 nM, 30 minutes). Cells were then fixed using 4 % formaldehyde and permeabilized with 0.1 % TX-100 in PBS. The cells were stained with 'Cell Painting'

assay reagents[1]: Hoechst 33342 (10 mg/mL stock, Molecular Probes, 1:5000), SYTO14 (Invitrogen, S7576, 3 µM), concanavalin A 488 (Invitrogen C11252, 1:150), Phalloidin-594 (Abcam, ab176757, 1:1500) and Wheat Germ Agglutinin 594 (Invitrogen, W11262) in 1% BSA/PBS. The plates were imaged using an ImageXpress high content microscope (Molecular Devices). Cells were imaged with a 20× objective, covering 6 fields-of-view in each well. The data was then analyzed using a custom CellProfiler software pipeline using the University of Edinburgh high performance computing cluster. Dose response plots were calculated using HC StratomineR (corelifeanalytics.com). Nuclei count data was analyzed separately to generate cell survival metrics (normalized to aqueous glycerol/TX-100 control). Dose response curves were fitted and IC_{50} values calculated using Prism GraphPad (graphpad.com). The 3D PCA plot in Figure 1 was generated from CellProfiler data using the Phenonaut software package.

Abbreviations

BSA, Bovine serum albumin; Boc, tert-butyloxycarbonyl; DAD, Diode array detector; DCM, Dichloromethane; DIPEA, N,N-Diisopropylethylamine; DMF, Dimethylformamide;; FC, Fraction Collector; Fmoc, Fluorenylmethyloxycarbonyl; HATU, Hexafluorophosphate Azabenzotriazole Tetramethyl Uronium; HPLC, High-Performance Liquid Chromatography; MWD, Multiple Wavelength Detector; PBS, phosphate buffer saline; Pbf, 2,2,4,6,7-pentamethyldihydrobenzofuran-5-sulfonyl; RPPA, Reverse Phase Protein Array; TCEP, tris(2-carboxyethyl)phosphine; Trt, trityl;

Monitoring of mitotracker intensities

The Cell Painting assay includes the mitochondrial visualization stain mitotracker. Image featurization and aggregation at the well level, followed by standardization to median DMSO control features on a plate-by-plate basis produces the

"MedianNucleiIntensityMADIntensityW5" feature describing the median well level intensity of the median absolute deviation the mitotracker stain across the nuclei. Similarly, the "MedianCellsIntensityMeanIntensityW5" feature summarizes the mean stain intensity over whole cells and reports this as a median at the well level. Figures S5 and S6 display these features after treatment with the Peptide 2 nullomer and Peptide 15 control peptide.

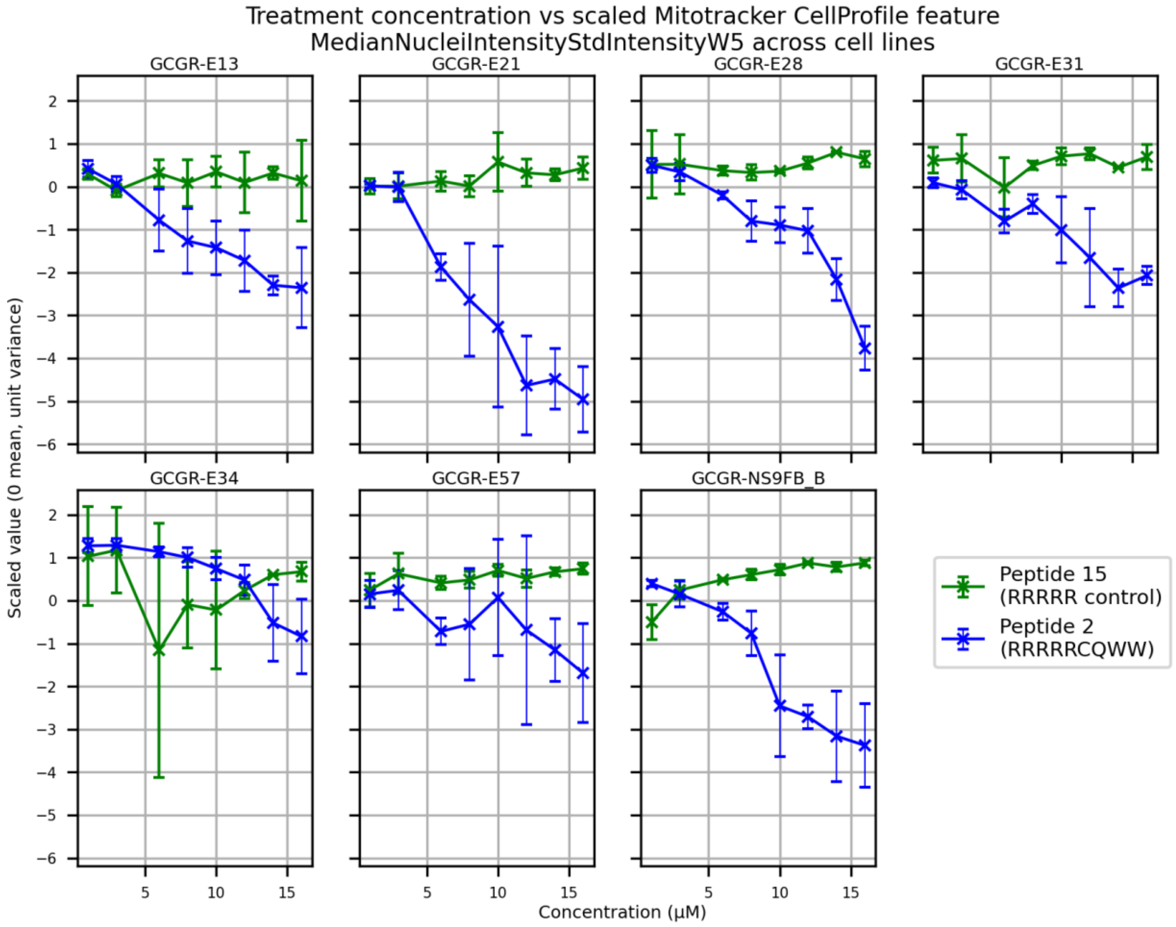

Figure S5 – The CellProfiler-derived feature "MedianNucleiIntensityMADIntensityW5", reading out mitotracker stain intensity observed in cell nuclei after treatment with the Peptide 2 nullomer (blue) and Peptide 15 (control). Values shown over all cell lines including the GCGR-NS9FB_B control. Addition of the nullomer peptide appears to reduce mitotracker intensity in a dose dependent manner.

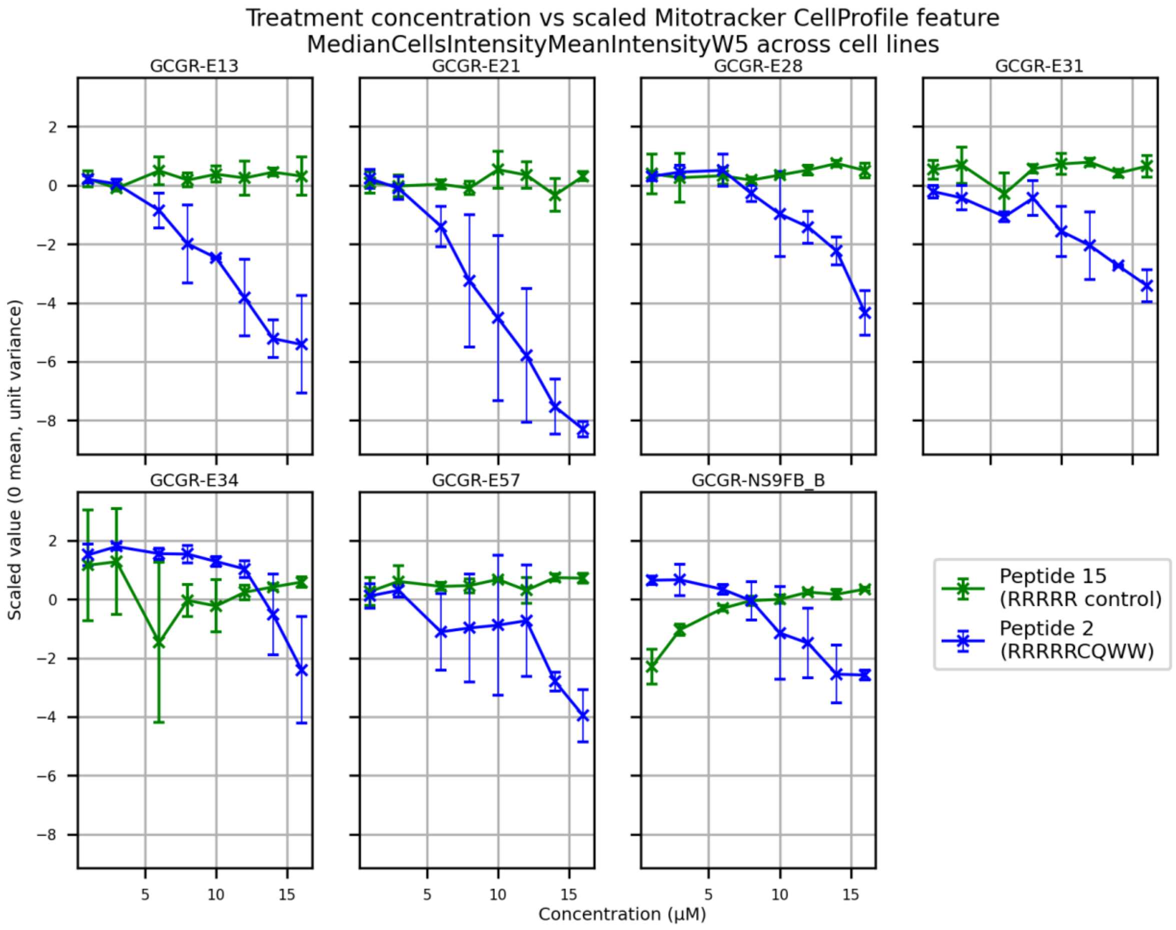

Figure S6 – The CellProfiler-derived feature "MedianCellsIntensityMeanIntensityW5", reading out mitotracker stain intensity observed in cells after treatment with the Peptide 2 nullomer (blue) and Peptide 15 (control). Values shown over all cell lines including the GCGR-NS9FB_B control. Addition of the nullomer peptide appears to reduce mitotracker intensity in a dose dependent manner.

References

(1) Bray, Mark-Anthony, et al. "Cell Painting, a high-content image-based assay for morphological profiling using multiplexed fluorescent dyes." *Nature protocols* 11.9 (2016): 1757-1774.
